# Evidence for Semantic Communication in Alarm Calls of Wild Sichuan Snub-Nosed Monkeys

**DOI:** 10.3390/biology14081028

**Published:** 2025-08-11

**Authors:** Fang-Jun Cao, James R. Anderson, Wei-Wei Fu, Ni-Na Gou, Jie-Na Shen, Fu-Shi Cen, Yi-Ran Tu, Min Mao, Kai-Feng Wang, Bin Yang, Bao-Guo Li

**Affiliations:** 1 Shaanxi Key Laboratory of Qinling Ecological Security, Shaanxi Key Laboratory for Animal Conservation, Shaanxi Institute of Zoology, Xi’an 710032, China; Fang198789@xab.ac.cn (F.-J.C.); wwfu2014@sina.com (W.-W.F.); gounina@ms.xab.ac.cn (N.-N.G.); wangkf@ms.xab.ac.cn (K.-F.W.); 2 Department of Psychology, Graduate School of Letters and Wildlife Research Center, Kyoto University, Kyoto 606-8501, Japan; jra6655@googlemail.com; 3 Research Center of Qinling Giant Panda, Shaanxi Academy of Forestry, Xi’an 710402, China; jienashen@sina.com; 4 College of Art, GuiZhou University of Finance and Economics, Guiyang 550025, China; cen248686@163.com (F.-S.C.); Tuyiran20250509@163.com (Y.-R.T.); 5 Department of Hotel Catering and Business Administration, Technological and Higher Education Institute of Hong Kong, Hong Kong 999077, China; 18821700069@163.com; 6 Shaanxi Key Laboratory for Animal Conservation, College of Life Sciences, Northwest University, Xi’an 710069, China; baoguoli@nwu.edu.cn

**Keywords:** *Rhinopithecus roxellana*, alarm calls, playback, referentiality, predators

## Abstract

The alarm calls of non-human primates help us to understand the evolution of animal vocal communication and the origin of human language. However, as there is a lack of research on alarm calls in primates living in multilevel societies, we studied these calls in wild Sichuan snub-nosed monkeys. By means of playback experiments, we analyzed whether call receivers understood the meaning of the alarm calls, making appropriate behavioral responses. Results showed that receivers made appropriate and specific anti-predator responses to two types of alarm calls. After hearing the aerial predator alarm call (“GEGEGE”), receivers’ first gaze direction was usually upward (towards the sky), and upward gaze duration was longer than the last gaze before playback. After hearing the terrestrial predator alarm call (“O-GA”), the first gaze direction was usually downward (towards the ground), and this downward gaze duration was longer than the gaze before playback. These reactions provide evidence for external referentiality of alarm calls in Sichuan snub-nosed monkeys, that is, information about the type of predator or the appropriate response is encoded acoustically in the calls.

## Simple Summary

The alarm calls of non-human primates have received extensive attention from researchers working on animal vocal communication. Through the use of an alarm call playback experiment, this study revealed that individual wild Sichuan snub-nosed monkey males who heard their species’ calls responded with appropriate anti-predator behaviors. In other words, the alarm calls were functionally referential. This study provides further information relevant to understanding the evolution of primate calls.

## 1. Introduction

Studies on the alarm calls of various animal species have shown that hearing alarm calls alone—even in the absence of other relevant information—can trigger appropriate predator-avoidance behaviors in the call receivers, similar to those shown toward a real predator [1,2,3]. These kinds of signals are referred to as “functionally referential” signals; that is, a “referent” (referring to the type of predator) generates a representation of that particular natural concept [1,4,5]. For instance, in the alarm call system of various monkeys, the vocal repertoire contains multiple acoustically distinct alarm calls, each one associated with the detection of a specific predator type (e.g., python, eagle, or leopard) [1,4]. Playback experiments have shown that these different alarm calls trigger specific behavioral reactions, which are usually adapted to the hunting methods of the predator, as if the monkeys hearing the calls actually saw the predators. For example, when hearing the alarm call for a raptor, monkeys run into dense vegetation, and on hearing the alarm call for a terrestrial predator, they are more likely to run and climb trees [1,4,6,7]. Although multiple studies show that some species have referential alarm calls for aerial versus terrestrial predators (e.g., see above, and for *Hylobates lar*, [8], others may not (e.g., *Saguinus mystax*, [9]). In some species, similar behavioral responses may be elicited by alarm calls for aerial predators and general, non-specific disturbances (e.g., *Sapajus apella*; [10]).

Based on research to date, the primate alarm call system is divided into three categories [4]: functional reference systems (e.g., *Saguinus fuscicollis*: [9]), causing receivers to respond differently for raptors and terrestrial predators; urgency functional reference systems (e.g., *Cercopithecus nictitans martini*: [11]), with graded signals reflecting the urgency of the response; and mixed functional reference systems (e.g., *Sapajus nigritus*: [10]), containing functionally referential and other, non-specific calls. The diversity of alarm call systems across species appears related to the fact that different predators elicit different kinds of defensive actions [12,13]. However, despite numerous studies on primate alarm calls, primates living in multi-level societies have been neglected. To broaden the range of species and social systems represented in this research area, we studied the Sichuan snub-nosed monkey, a largely arboreal, multi-level social primate [14,15]. As social complexity may promote the evolution of vocal complexity [16], research on alarm calls in multi-level social primates can shed further light on call evolution [17].

The Sichuan snub-nosed monkey (*Rhinopithecus roxellana*) is an endangered colobine primate endemic to the Qinling Mountains of Shaanxi Province, China [14,18]. Studies on this species’ vocal communication have revealed a variety of vocalizations that include an alarm call (O-GA) for ground-level disturbances, humans, dogs, etc. Long-term observations and recordings have also confirmed another alarm call (GEGEGE), emitted when the monkeys detect a potential aerial predator. At present, however, the precise functions and referentiality of the alarm calls of Sichuan snub-nosed monkeys are still unclear. We conducted playback experiments to test whether acoustic information alone is sufficient to elicit predator-specific reactions in these monkeys. Based on reactions observed during natural predator encounters, we made the following predictions: (1) In response to GEGEGE alarm call playback, the focal receiver would look mostly upwards. (2) In response to O-GA alarm call playback, the focal receiver would look mostly downwards. (3) In response to GEGEGE alarm calls, monkeys would scan the sky and avoid remaining in exposed areas. (4) In response to O-GA alarm calls, monkeys would scan the ground and orient towards or approach the playback speaker, to obtain information about the reason for the call and potentially to mob the predator, a behavior often observed during terrestrial predator encounters.

## 2. Materials and Methods

### 2.1. Study Site and Species

Playback experiments were performed on two groups of Sichuan snub-nosed monkeys, located in Dapingyu (33°40′25.1688″ N, 107°59′0.0204″ E), Foping County; and Huangguan Town (33°37′55.7112″ N, 108°22′52.2984″ E), Ningshan County, in the Qinling Mountains, Shaanxi Province, China [18,19]. Groups usually consist of several one-male units (OMUs) and all-male units (AMUs). A typical OMU includes one adult male, several adult females, and several juveniles and infants, while an AMU is composed of an adult male and one or more sub-adult males [18,19]. The studied groups have been continuously under observation since 2009 and 2010, respectively. During this study, the Huangguan group had approximately 110–130 members, and the Foping group had 70–90 members. To obtain better observation conditions, food provisioning was started on 20 April 2010; a research assistant scattered a small amount of corn, apple slices, and carrot slices at a fixed location in the valley at 09:00, 12:00, and 15:00 every day. Otherwise, to minimize influencing the behavior of the monkeys, the amount of food given was fixed and limited; the monkeys were always free to roam and forage on natural foods. Researchers can observe these monkeys from as close as 2 m up to 50 m. All individuals are identifiable based on sex, body size, coat color, and idiosyncratic physical features [15,19]. Monkeys were classified into four age/sex categories: adult males and females (5 years and above), juveniles (1 to 5 years), and infants (under 1 year) [14].

### 2.2. Data Collection and Playback Stimuli

**Figure 1:**
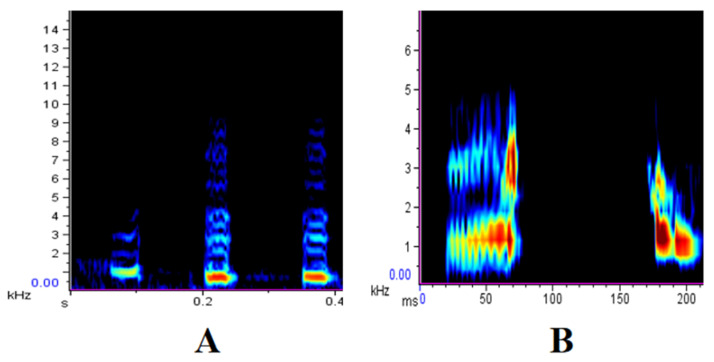
Alarm calls of the Sichuan snub-nosed monkey. (**A**) GEGEGE call; (**B**) O-GA call.

The alarm calls used in the playback experiments originated from the two study populations, i.e., the Huangguan population (recorded from February to December 2018) and the Foping population (recorded from September 2024 to January 2025). All alarm calls occurred naturally in the wild as responses to predators. The two types of calls used as playback stimuli were the GEGEGE call (emitted when encountering aerial raptors) (Figure 1A) and the O-GA call (emitted in response to terrestrial predators) (Figure 1B). Call recordings were obtained using Sennheiser K6/M66 directional microphones and a MARANTZ PMD660 solid-state recorder (the recording settings were 44.1 kHz sampling rate; 16-bit accuracy). Sounds selected for playback experiments were of high quality, with no noise or other disturbances, and were emitted by different callers [20].

Thirty AMU males were selected as subjects for the playback experiment. Controlling the influence of social relationships, the alarm caller identities (playback) were all unfamiliar to AMU males (compared to the familiar individuals within the OMU). Each individual was played two different alarm calls, each from different individuals belonging to the same group. Only one alarm call-type playback test was broadcast on any given day to avoid habituation, and there was an interval of at least 5 days between playbacks of the same kind. To avoid interference from other individuals, subjects were tested only when at least 30 m away from the group (sitting quietly on the ground and eating), and the sound source came from the last-known direction of the group (playback from a realistic location, that is, the location of the other individuals). To prevent any possible impact, intervals of at least 2 days separated the tests on different individuals. Only playback tests without any disturbance from other individuals, predators, or humans were used for analyses; disturbed tests were halted and reconducted at least 30 min later. The responses of the subjects (focal receivers) to playbacks were recorded on video. The stimuli were broadcast through an Apple iPod Nano, connected to a Sony SRS-XB43 speaker hidden in bushes (to avoid attracting monkeys’ attention), 10 cm above the ground, about 10 m away from the subject, and at a 90-degree angle to the subject. Using an adjustable iPod volume control, playback stimuli were broadcast within the monkeys’ natural amplitude range, with care taken to make them sound as natural as possible to a human at a distance of about 10 m [20].

We used focal animal sampling [21] and all-occurrence recording [22,23] to record behavioral responses to the playbacks. Along with subject identity and type of alarm call, we recorded the subject’s first gaze direction and bodily movement, as well as downward and upward gazes before and after the playback. Gaze directions were classified as follows: (1) “Looking upwards towards the sky”, with the head estimated to be at least 45 degrees above horizontal; (2) “Looking downwards towards the ground”, with the head estimated to be at least 45 degrees below horizontal; (3) “Towards the speaker”, with the head within 45 degrees relative to the axis formed with the speaker. Locomotor responses were classified as: (1) not move; (2) upwards (moving towards higher ground, bushes, or trees, or climbing); (3) downwards (moving towards lower ground, or descending bushes or trees); (4) towards the speaker.

### 2.3. Statistical Analysis

Statistical analyses were performed using IBM SPSS Statistic 23 software and GraphPad Prism 9.0. Normality was assessed using the Shapiro–Wilk test. For categorical data, we conducted Chi-squared tests. As the gaze duration data followed a normal distribution, a paired t-test was used to compare gaze durations before and after alarm call playback. A *p*-value of less than 0.05 was considered significant.

### 2.4. Ethics Statement

This research complies with the regulations of the China Wildlife Conservation Association regarding the ethical treatment of research subjects and also conforms to the laws of the People’s Republic of China concerning the protection of wild animals. The research was conducted entirely using noninvasive methods unlikely to have any impact on the welfare of the monkeys participating in the study.

## 3. Results

### 3.1. Gaze Duration

**Figure 2:**
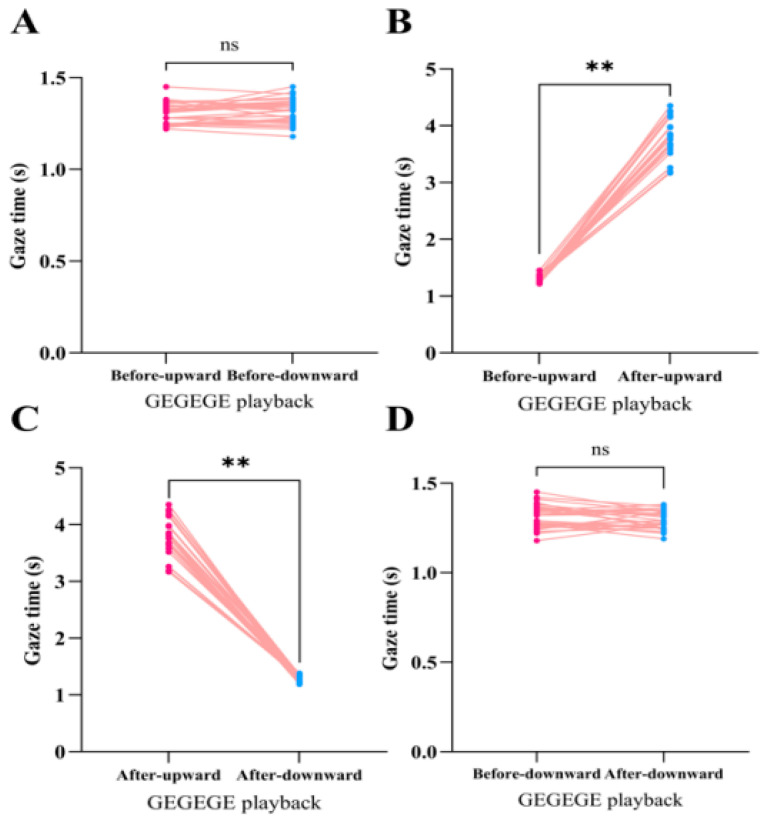
Gaze durations following GEGEGE alarm call playback. A paired t-test was used to compare gaze durations: (**A**) Before-upward vs Before-downward; (**B**) Before-upward vs After-upward; (**C**) After-upward vs After-downward; (**D**) Before-downward vs After-downward. The red and blue dots represent the gaze duration and pink lines represent individual. ns: *p* > 0.05, **: *p* < 0.01.

Mean gaze durations before and after playback of the GEGEGE alarm call are shown in Figure 2. Before playback, there was no significant difference between subjects’ downward and upward gaze durations (Paired t test, t(29) = 1.54, *p* = 0.14 > 0.05; Figure 2A). Upward gaze duration after playback was longer compared to that before playback (t(29) = 37.97, *p* < 0.01; Figure 2B), and longer than the post-playback downward gaze duration (t(29) = 39.43, *p* < 0.01; Figure 2C). However, downward gaze duration after playback was not significantly different from that before playback (t(29) = −1.80, *p* = 0.83 > 0.05; Figure 2D).

**Figure 3:**
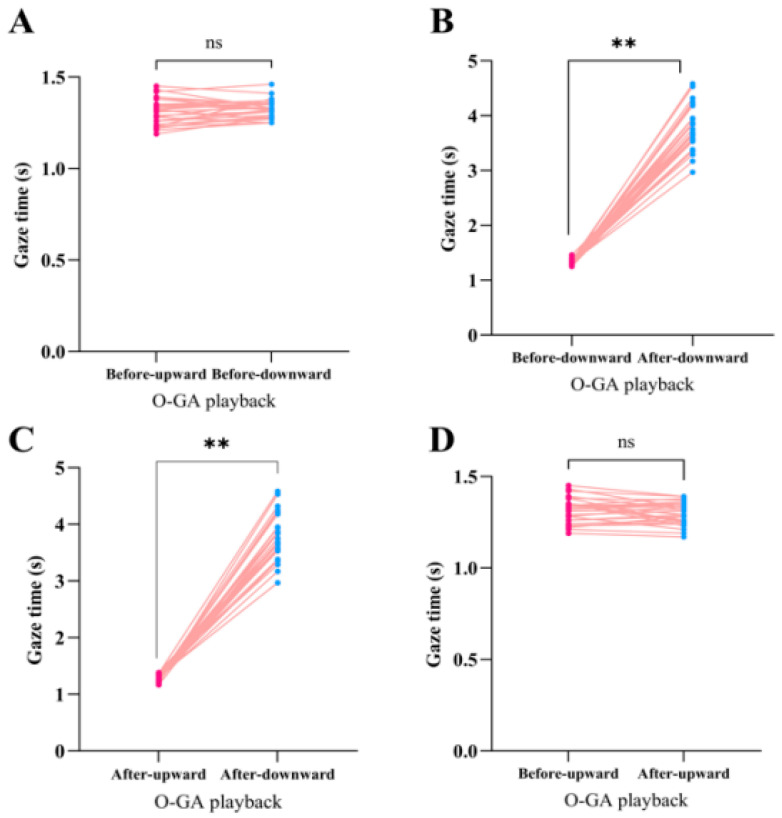
Gaze durations following O-GA alarm call playback. A paired t-test was used to compare gaze durations: (**A**) Before-upward vs Before-downward; (**B**) Before-downward vs After-downward; (**C**) After-upward vs After-downward; (**D**) Before-upward vs. After-upward. The red and blue dots represent the gaze duration and pink lines represent individual. ns: *p* > 0.05, **: *p* < 0.01.

Mean gaze durations before and after playback of the O-GA alarm call are shown in Figure 3. Before playback, there was no significant difference between downward and upward gaze durations (t(29) = 1.64, *p* = 0.11 > 0.05; Figure 3A). After playback, downward gaze duration was longer compared to before (t(29) = 34.43, *p* < 0.01; Figure 3B), and longer than post-playback upward gaze (t(29) = 33.84, *p* < 0.01; Figure 3C). However, post-playback upward gaze duration was not significantly different from the corresponding pre-playback duration (t(29) = −1.47, *p* = 0.15 > 0.05; Figure 3D).

**Figure 4:**
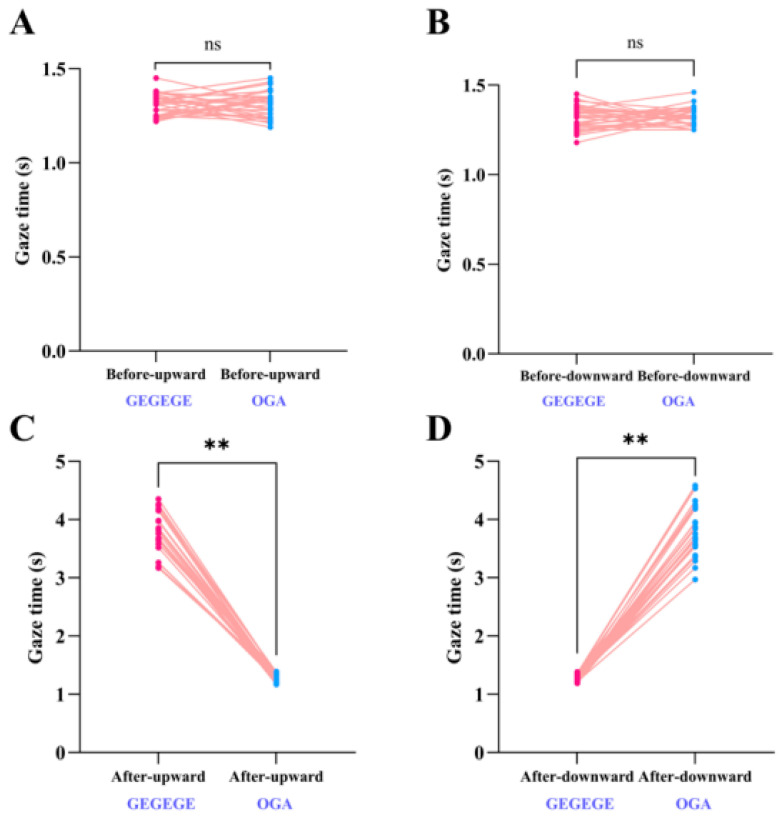
Gaze durations following GEGEGE and O-GA alarm call playback. A paired t-test was used to compare gaze durations: (**A**) Before-upward vs Before-upward; (**B**) Before-downward vs Before-downward; (**C**) After-upward vs After-upward; (**D**) After-downward vs After downward. The red and blue dots represent the gaze duration and pink lines represent individual. ns: *p* > 0.05, **: *p* < 0.01.

There was no significant difference in pre-playback upward gaze duration between GEGEGE and O-GA calls (t(29) = −0.50, *p* = 0.62 > 0.05; Figure 4A), nor did downward gaze durations differ significantly (t(29) = −0.65, *p* = 0.52 > 0.05; Figure 4B). By contrast, following playback, upward gaze duration was longer for GEGEGE calls than O-GA calls (t(29) = 39.90, *p* < 0.01; Figure 4C), while downward gaze duration was longer after an O-GA call than a GEGEGE call (t(29) = 37.95, *p* < 0.01; Figure 4D).

### 3.2. Direction of First Gaze

**Figure 5:**
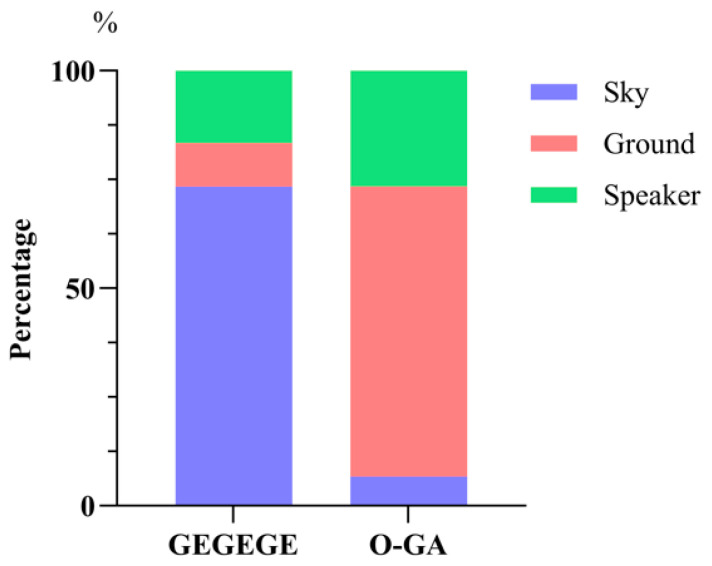
Direction of first gaze after playback of two types of alarm call.

Following playback of the GEGEGE alarm call, most monkeys’ first gaze was directed up towards the sky (73.33%, 22/30), followed by looking towards the speaker (16.67%, 5/30); a few individuals looked down towards the ground (10.00%, 3/30) (Figure 5). However, following playback of the O-GA alarm call, most monkeys first looked down towards the ground (66.67%, 20/30), followed by towards the speaker (26.66%, 8/30), with just two individuals looking up towards the sky (6.67%, 2/30). The difference in the first gaze direction following playback of the two types of alarm calls was significant (Chi-square test, χ2 (2) = 29.92, *p* < 0.01; Figure 5).

### 3.3. Locomotor Responses

**Figure 6:**
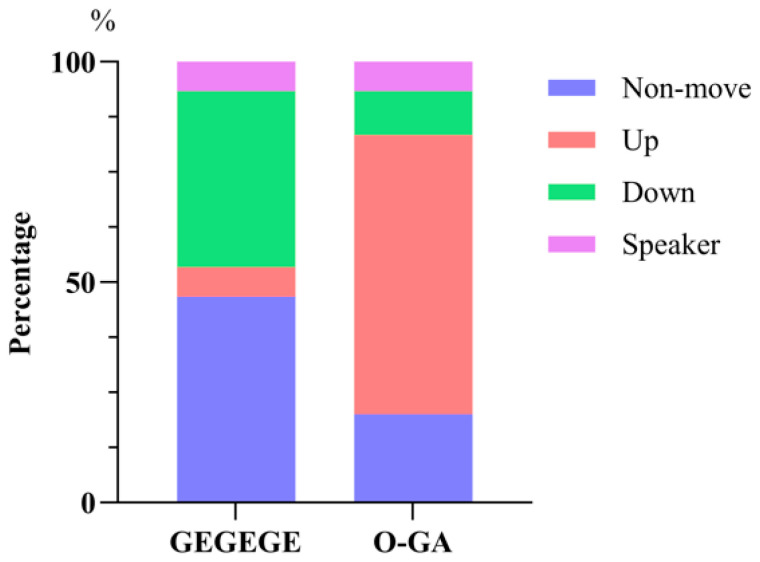
Direction of movement following playback of two types of alarm call.

After playback of the GEGEGE alarm call, almost half of the monkeys did not noticeably move location (46.66%, 14/30). Forty percent of them moved downwards (towards shrubs) (12/30), while just two moved up towards higher ground or trees (6.67%) or moved towards the speaker (6.67%, 2/30) (Figure 6). After playback of the O-GA alarm call, most monkeys moved upwards (63.33%, 19/30), 20% made no discernible movement (6/30), and just a few moved downwards (10.00%, 3/30) or in the direction of the speaker (6.67%, 2/30) (Figure 5). The differences in locomotor responses after the GEGEGE and O-GA alarm call playbacks were significant (Chi-square test, χ2 (3) = 22.36, *p* < 0.01; Figure 6).

## 4. Discussion

This is the first alarm call playback experiment on wild Sichuan snub-nosed monkeys. The monkeys’ reactions to the broadcast alarm calls were related to the type of predator that originally elicited the calls, or at least its perceived location. The two types of alarm calls indicate different external objects or events to those who hear them. Following playback of the aerial predator alarm call (GEGEGE), upward gaze duration was longer than before the playback, and also longer than post-playback downward gaze duration. By contrast, the terrestrial predator alarm call (O-GA) elicited significantly longer downward gaze compared to before playback and compared to post-playback upward gaze. Furthermore, upwards gaze duration was longer for GEGEGE than for the O-GA alarm, whereas the converse was found for downwards gaze. Different alarm calls elicited different visual orienting by the receivers, suggesting that the latter obtained information about at least the general location of the source of the danger. This is similar to the results of playback experiments on other primate species [7,10,24,25].

Upon hearing the GEGEGE alarm call, most of the Sichuan snub-nosed monkeys’ first gaze was directed upwards towards the sky, whereas the O-GA alarm call caused most of them to first look downwards towards the ground. These results suggest that monkeys who hear them can distinguish the type of threat represented by the alarm calls, leading to appropriate orienting responses, as previously described in other monkeys [1,26]. Also, like some other species, the Sichuan monkeys look in the direction of the caller (the speaker), presumably to obtain more information about the danger [24,27]. Combined with naturalistic field observations, our experiment confirms that the GEGEGE alarm call indicates danger from the air, i.e., raptors; it is not emitted for other kinds of disturbance. In contrast, the O-GA call is a general alarm call, emitted upon detection of terrestrial predators as well as non-predatory disturbances on the ground. Notably, playback of the O-GA call causes the receiver to orient towards the speaker, as if searching for additional information (e.g., the caller’s behavior or direction, see [7,11,25,27,28]). Given the diversity of scenarios that elicit the O-GA alarm call, the latter can be defined as “a generalized terrestrial disturbance call” rather than a functionally referential call, as it does not seem to refer to a single entity or event [25,29]. Our findings are consistent with other studies in which predator-related calls were sometimes given to non-predator events; although these calls often elicit predator-appropriate responses, their environmental specificity level is lower [9,29,30].

Finally, we found that after hearing the GEGEGE alarm call, most of the receivers did not move, while some moved downward, in the direction of shrubs. Upon hearing the O-GA alarm call, most moved up towards the trees, while a few others remained stationary, indicating that the monkeys’ defensive reactions differ with the kind of alarm call emitted. After hearing the alarm call for raptors (GEGEGE alarm call), monkeys usually scanned the tree canopy or the sky and either froze or quickly fled, usually downward to a place offering more overhead protection. The (O-GA) alarm call used for terrestrial predators usually resulted in scanning the ground or the lower canopy, locating and approaching the caller, and often gathering to harass the predator, similar to reactions described in other primates [7,25,30].

## 5. Conclusions

In conclusion, our playback experiment on responses to alarm calls of Sichuan snub-nosed monkeys showed that hearers of alarm calls attribute different meanings to the calls, even in the absence of real predators or companions, and employ appropriate visual orienting responses and risk-minimizing strategies. These findings strongly suggest that alarm call receivers can infer specific, possibly relevant information on external events contained in these calls [4]. Their responses are tailored to potential external referents, that is, to information about the type of predator or the appropriate reaction, encoded in the acoustic characteristics of the calls [4,7,25]. The alarm calls of the Sichuan snub-nosed monkeys have, therefore, evolved to have functional referentiality [5,7,25]. This study combined taking into consideration the social relationships of a multi-level social primate while excluding the interference of specific social relationships on individual monkeys’ understanding of alarm calls. For a more complete picture of the evolution of alarm calls and the responses they elicit, more comparative studies of primates with different social structures are required. During our ongoing research on alarm calls of Sichuan snub-nosed monkeys, we will continue to record natural disturbances and conduct experiments that will incorporate realistic predator models. We also plan to examine the influence of age, sex, and social relationships on the use and understanding of alarm calls.

## Data Availability

Data are available upon request.
