# Correction: Cao et al. Evidence for Semantic Communication in Alarm Calls of Wild Sichuan Snub-Nosed Monkeys. *Biology* 2025, *14*, 1028

**DOI:** 10.3390/biology14091139

**Published:** 2025-08-29

**Authors:** Fang-Jun Cao, James R. Anderson, Wei-Wei Fu, Ni-Na Gou, Jie-Na Shen, Fu-Shi Cen, Yi-Ran Tu, Min Mao, Kai-Feng Wang, Bin Yang, Bao-Guo Li

**Affiliations:** 1 Shaanxi Key Laboratory of Qinling Ecological Security, Shaanxi Key Laboratory for Animal Conservation, Shaanxi Institute of Zoology, Xi’an 710032, China; 2 Department of Psychology, Graduate School of Letters and Wildlife Research Center, Kyoto University, Kyoto 606-8501, Japan; 3 Research Center of Qinling Giant Panda, Shaanxi Academy of Forestry, Xi’an 710402, China; 4 College of Art, GuiZhou University of Finance and Economics, Guiyang 550025, China; 5 Department of Hotel Catering and Business Administration, Technological and Higher Education Institute of Hong Kong, Hong Kong 999077, China; 6 Shaanxi Key Laboratory for Animal Conservation, College of Life Sciences, Northwest University, Xi’an 710069, China

## Error in Figure

In the original publication [1], there was a mistake in Figure 1 as published. Subfigure A and B from Figure 1 were reversed. The corrected Figure 1 appears below. The authors state that the scientific conclusions are unaffected. This correction was approved by the Academic Editor. The original publication has also been updated.

## Figures and Tables

**Figure 1:**
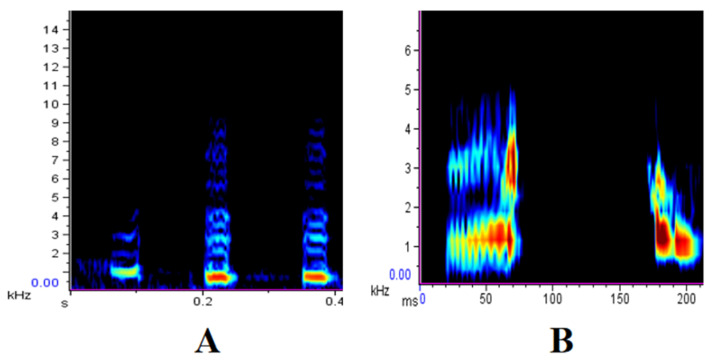
Alarm calls of the Sichuan snub-nosed monkey. (**A**) GEGEGE call; (**B**) O-GA call.
